# General deep learning model for detecting diabetic retinopathy

**DOI:** 10.1186/s12859-021-04005-x

**Published:** 2021-11-08

**Authors:** Ping-Nan Chen, Chia-Chiang Lee, Chang-Min Liang, Shu-I Pao, Ke-Hao Huang, Ke-Feng Lin

**Affiliations:** 1 Department of Biomedical Engineering, National Defense Medical Center, Taipei, 114 Taiwan, ROC; 2 Graduate Institute of Applied Science and Technology, National Taiwan University of Science and Technology, Taipei, 106 Taiwan, ROC; 3 Department of Ophthalmology, Tri-Service General Hospital, National Defense Medical Center, Taipei, 114 Taiwan, ROC; 4 Department of Medical Records, Tri-Service General Hospital, National Defense Medical Center, Taipei, 114 Taiwan, ROC

**Keywords:** SMOTE, Overfitting, Decision tree, Nasnet-large, Transfer learning

## Abstract

**Background:**

Doctors can detect symptoms of diabetic retinopathy (DR) early by using retinal ophthalmoscopy, and they can improve diagnostic efficiency with the assistance of deep learning to select treatments and support personnel workflow. Conventionally, most deep learning methods for DR diagnosis categorize retinal ophthalmoscopy images into training and validation data sets according to the 80/20 rule, and they use the synthetic minority oversampling technique (SMOTE) in data processing (e.g., rotating, scaling, and translating training images) to increase the number of training samples. Oversampling training may lead to overfitting of the training model. Therefore, untrained or unverified images can yield erroneous predictions. Although the accuracy of prediction results is 90%–99%, this overfitting of training data may distort training module variables.

**Results:**

This study uses a 2-stage training method to solve the overfitting problem. In the training phase, to build the model, the Learning module 1 used to identify the DR and no-DR. The Learning module 2 on SMOTE synthetic datasets to identify the mild-NPDR, moderate NPDR, severe NPDR and proliferative DR classification. These two modules also used early stopping and data dividing methods to reduce overfitting by oversampling. In the test phase, we use the DIARETDB0, DIARETDB1, eOphtha, MESSIDOR, and DRIVE datasets to evaluate the performance of the training network. The prediction accuracy achieved to 85.38%, 84.27%, 85.75%, 86.73%, and 92.5%.

**Conclusions:**

Based on the experiment, a general deep learning model for detecting DR was developed, and it could be used with all DR databases. We provided a simple method of addressing the imbalance of DR databases, and this method can be used with other medical images.

## Background

Patients with diabetes visit the hospital when their eyesight worsens. They often have proliferative diabetic retinopathy (DR) or vitreous hemorrhage. Currently, doctors can detect symptoms early by using retinal ophthalmoscopy, and they can improve diagnostic efficiency by using deep learning techniques to select treatments and support personnel workflow [1].

The determination of retinopathy severity requires extensive professional knowledge. Depending on physician experience, interpretations of the same data set can vary, causing errors. Therefore, images must be quantified. The comparison of preoperative and postoperative data can help physicians judge whether an operation is successful. In addition, diagnoses can be confirmed using machine learning and deep learning and required treatments can be clearly identified. These techniques can help physicians make accurate diagnoses and identify lesions [2, 3].

The results obtained from optical coherence tomography, ophthalmoscopy, and the automatic classification of multicategorical anomalies based on deep learning are similar to those obtained from clinical classification, the accuracy of these imaging methods is only 70–85% [4–7]. A deep learning training model may have tens or hundreds of thousands of parameters. Small training data sets may affect the model’s diagnostic accuracy, especially when data sets with unbalanced classifications are used. In 2015, EyePACS and the California Healthcare Foundation organized a competition for data scientists on the Kaggle platform [8]. DR data used were divided into five categories: 0 (no DR), 1 (mild DR), 2 (moderate DR), 3 (severe DR), and 4 [proliferative DR (PDR)]. The training image distribution ratio used in the competition was 25,810 to 2443 to 5292 to 873 to 708. This is a typical case of unbalanced data, meaning that as long as the prediction result is “no DR,” the model’s final diagnostic accuracy is 73% [9].

## Related work

Abnormal samples in medical images are less common than normal samples and often valuable; therefore, studying unbalanced data is crucial. Unbalanced data can be addressed using oversampling (e.g., the synthetic minority oversampling technique (SMOTE)) [10, 11], under sampling [12], weighted sampling [13], and cross-validation (e.g., k-fold cross-validation) [14–16]. In k-fold cross-validation, training data are divided into k equal parts. Each time, only one aliquot is used as the test data set, and the remaining data are used as the training set. Gradually, each aliquot is used as the test dataset, and the k average is used as the final accuracy of the database. It was concluded that when the data are divided into five equal parts, and the model’s accuracy rate can reach 92.24% [16]. Another deep learning method of preventing data imbalance is random oversampling, which increases the number of training images through the translation, rotation, deformation, scaling, and noise addition by using a small set of images. In this method, the number of classification libraries is balanced, which increases the accuracy of the deep learning model. For example, 16,500 digital images were generated using 9316 images from the Kaggle competition database through oversampling, and an accuracy rate of 86.1% was obtained through k-fold cross-validation [13]. Another study employing the Kaggle competition database screened 10,000 images of specific differences to perform training verification and prediction; 99.05% accuracy was achieved [17]. Another study used the Diabetic Retinopathy Images Database. Of 216 images in the database, 125 were good, 69 were poor, and 22 were abnormal during deep learning training, and the prediction accuracy rate was 99.98% [18]. Other methods of addressing data imbalance in deep learning (such as undersampling) are rarely used. The detection rate of DR with or without exudate classification reached 98% in a study [19]. Most articles divide the data set into a learning subset and a test subset (80% and 20% respectively), and then use techniques such as subsampling or synthetic secondary oversampling (SMOTE) for resampling, in order to obtain a perfectly balanced training set, and achieve a prediction accuracy of 93–99% [20–22].

The aforementioned methods for processing unbalanced data can produce high diagnostic rates. When the parameters of the deep learning model are sufficiently complex, the training model will record the features of all pictures in the training data set, so untrained samples usually lead to recognition errors. Therefore, some scholars pointed out that oversampling may lead to overfitting of the model [19, 20].

There are methods to avoid overfitting, such as: (a) collecting more data: more data can help models training better; (b) stopping training early: when the validation loss rises, you can stop training immediately. Because after that, more training may make the model worse; (c) normalizing L1 and L2: prevent the model from being affected by the parameters with higher weight coefficients, which will lead to overfitting; (d) dividing data set: divide data sets into three groups, keep the holdout data as the test set to estimate the generalization performance of model. In this study, we apply the stopping training early and dividing the dataset into three parts to avoid the overfitting.

**Fig. 1:**
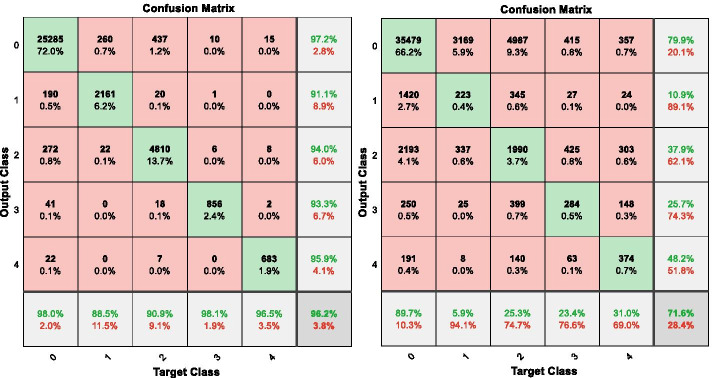
The confusion matrix on the training set (left) and test set (right) of the EyePACS data set using the pre-trained AlexNet model with DR category

To prove that oversampling will lead to overfitting of the model. We apply the SMOTE algorithm to synthesize several types of images in the open training database of the Kaggle competition, and we trained and validated datasets at 80:20 training-to-validation set ratio; then, we fine-tuned a pretrained AlexNet [23, 24] convolutional neural network (CNN) to classify these datasets. The final trained network achieved 96.2% accuracy for the trained and validated set and the confusion matrix in Fig. 1 (left). It indicates that nearly all DR classifications achieved more than 90% accuracy. Classifications of 0 (no DR) and 3 (severe DR) achieved 98% accuracy. However, when this training model classified untrained images (53,576), its accuracy was only 71.6% and the confusion matrix of this scenario is presented in Fig. 1 (right). For categories other than 0 (no DR), the accuracy rate was only 5.9–31.0%. This huge gap between trained model and test set show the result of overfitting from oversampling.

This study used the classic method of preventing overfitting by dividing the data sets into three groups (i.e., training, validation, and test sets) and used a two-stage training approach to mitigate overfitting caused by SMOTE. Subsequently, we provide a complete description of the DR databases and experimental data used to validate our proposed method.

## Methods

### Data, hardware, and software

We used six public datasets for model training and testing. The EyePACS [25] dataset was applied with pre-trained NASNet-Large [26] to tune the hyper-parameters. The DIARETDB0 [27], DIARETDB1 [28], eOphtha [29], MESSIDOR [30], DRIVE [31], were used to evaluate the model’s performance.

EyePACS is a free platform that includes retinopathy images. A clinician rated DR in each image on a scale of 0–4, where 0 indicates no DR, 1 indicates mild DR, 2 indicates moderate DR, 3 indicates severe DR, and 4 indicates PDR. In our study, images with classifications of 0, 1, 2, 3, and 4 were represented by 65,343, 6205, 13,153, 2087, and 1914 training samples, respectively.

The DIARETDB0 data set consisted of fundus images. We tested their algorithms by using 130 image communities capture using a 50° field of view. Of these images, 110 presented signs of DR, and 20 were normal.

The DIARETDB1 database were captured using a 50° field of view. Of these images, 5 were normal and 84 presented signs of DR.

The eOphtha is a free database that provides color fundus images, of which 47 had exudates and 35 were normal.

The MESSIDOR (Methods to Evaluate Segmentation and Indexing Techniques in the field of Retinal Ophthalmology) data set includes 1251 digital color images of the posterior pole obtained using a Topcon TRC NW6 nonmydriatic retina camera with three color charge-coupled devices. Images were captured at resolutions of 1440 × 960, 2240 × 1488, or 2304 × 1536 pixels using an 8-bit color plane.

The DRIVE (Digital Retinal Images for Vessel Extraction) [31] data set was established to facilitate comparative research on blood vessel segmentation in retinal images. Retinal images were obtained from a Dutch DR screening program. The screening population consisted of 400 patients with diabetes aged 25–90 years. Of 40 randomly selected images, 33 did not present signs of DR, and 7 presented signs of early, mild DR.

**Fig. 2:**
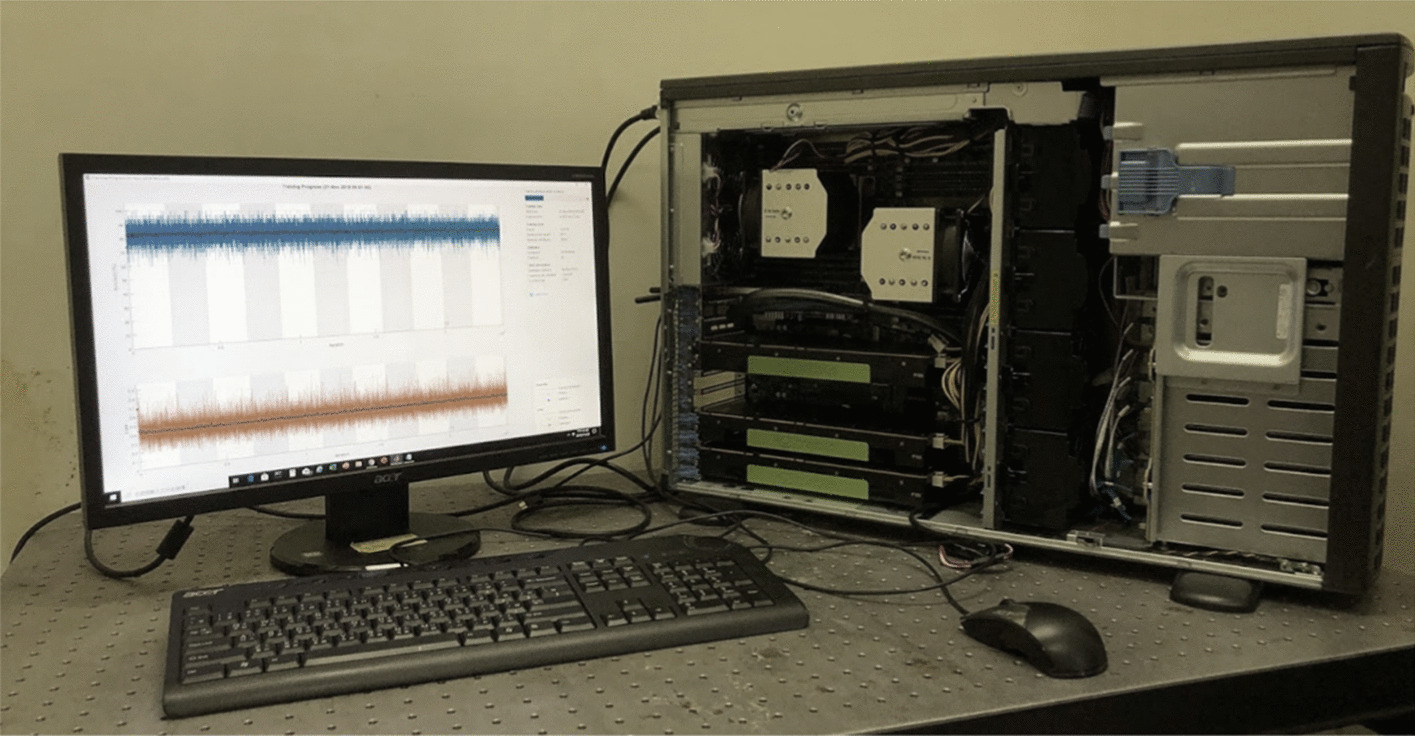
Deep learning machine

The deep learning machine presented in Fig. 2 with the following specifications was used to train our model:

- Workstation: Supermicro GPX XS8-24S1-4GPU; central processing unit: Intel Xeon Platinum 8165 processor *2; random-access memory: 128-GB DDR4 ECC 2666; graphics processing unit (GPU), 16-GB NVIDIA TESLA P100 *3.
- Operating system and software: Windows 10 (64 bit), Matlab2019a Deep Learning Toolbox, Compute Unified Device Architecture (CUDA) Toolkit 10.1, CUDA Deep Neural Network v7.6.0 (May 20, 2019).

### Methodological overview

We divided all the data sets into two parts, the training phase used the EyePACS dataset; and the test phase used the DIARETDB0, DIARETDB1, eOphtha, MESSIDOR, and DRIVE datasets.

In training phase, the main cause was to build the Diabetic Retinopathy detection model, the Deep Learning module 1 used the preprocessed EyePACS dataset and pre-trained NASNET-Large to identify the DR and no-DR. The Deep Learning module 2 apply the SOMTE synthetic samples to identify the mild-NPDR, moderate NPDR, severe NPDR and proliferative DR. When validation loss rises, these 2 modules stop training immediately to perform the hyperparameters tuning then restart the training to avoid the model worse.

**Fig. 3:**
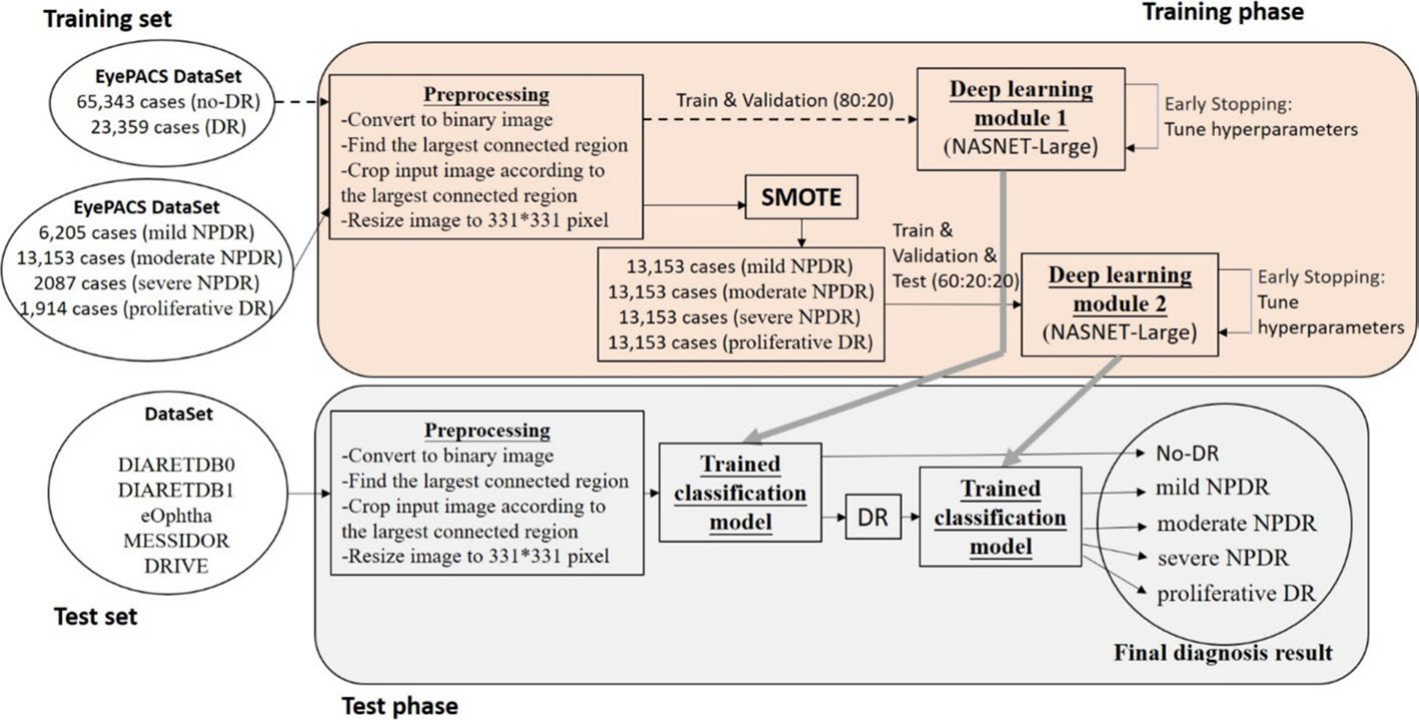
Flowchart of classifying DR severity into five stages

In test phase, the test data sets input the Deep Learning module 1 to identify DR and no-DR categories, then the DR images use Deep Learning module 2 for the classification to mild-NPDR, moderate NPDR, severe NPDR and proliferative DR. The overall system flowchart for classifying DR categories is presented in Fig. 3.

#### Step 1: Preprocessing

*Normalization of fundus images*: The resolutions of the original fundus images were inconsistent (e.g., 4752 × 3168, 3888 × 2592, and 2592 × 1944 pixels). However, if images were adjusted to a uniform 331 × 331-pixel resolution, the eyeball sphere would be deformed, resulting in low-resolution images in deep learning training. Therefore, we first converted color images into grayscale through binarization. Subsequently, connected-component labeling was performed to mark all connected areas of the image and identify the largest connected area, which was focused on the eyeball in the fundus images. Finally, the original color images were cropped according to the range of the maximum connected region to obtain images cropped at the edges of the eye.

*Pretrained deep neural network selection*: Transfer learning was used to establish a diagnostic model for medical imaging analysis, and we selected the pretrained NASNet-Large CNN to classify our images. According to MATLAB 2019a documentation [32], transfer learning with the pretrained NASNet-Large CNN can achieve 85% classification accuracy in 16 training modules. Therefore, this study used this NASNet-Large pretrained learning module and normalized the resolution of the fundus images to 331 × 331 pixels.

#### Step 2: Deep learning module

*Unbalanced data processing*: Traditional SMOTE data processing involves the rotation, scaling, and translation of training images to increase the number of training samples. SMOTE may cause variable importance distortion in the training module. Therefore, SMOTE was not used in the first step of deep learning training to address data imbalance. We first used a decision tree and NASNet-Large transfer learning to initially classify DR into no-DR (65,343 sheets) and DR (23,359 sheets) categories. Second, the DR category was classified into four categories based on NPDR and PDR. Finally, the five-stage classification of DR severity was used in combination with the two aforementioned diagnostic models.

### Fine tuning

**Fig. 4:**
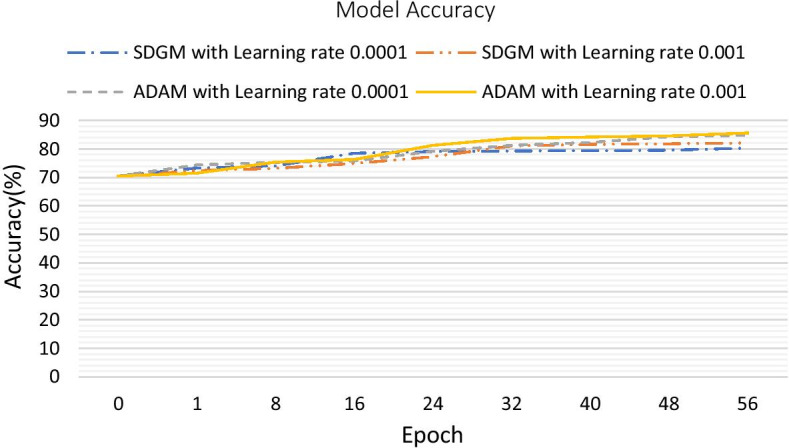
Model accuracy with different optimization algorithms and learning rates

In hyperparameter adjustment, this research applies 2 phase experiment. First, execute the 56 epochs to separate the performance of SDGM and ADAM under the learning rate by 0.0001 and 0.001. Figure 4 shows the accuracy of models with different optimization algorithms and learning rates. Therefore, we set the optimization algorithm as ADAM, and the learning rate is 0.001 as the parameters of deep learning model. Second, apply ADAM with learning rate 0.001 for the 2 days period training as the minibatch value turning. If the performance drops, adjust the minibatch value downward (32, 24, 16, 12, 8). In the end we find out the best parameters for the model.

## Results

This study used the classic method of preventing overfitting by dividing the data sets into three groups (i.e., training, validation, and test sets) and used a two-stage training approach to mitigate overfitting caused by SMOTE.

### 2-stages method (this study proposed)

We used the NASNet-Large deep CNN to pretrain the model, which contained EyePACS images with no DR (65,343 sheets) and DR (23,359 sheets). The Deep Learning module 1 achieved an accuracy of 0.85 in terms of no DR and DR classification. The combined Deep Learning module 1 and Deep Learning module 2 trained network had an accuracy of 0.83 in classifying DR into five categories of severity (0, no DR; 1, mild NPDR; 2, moderate NPDR; 3, severe NPDR; and 4, proliferative DR). Finally, we used the DIARETDB0, DIARETDB1, eOphtha, MESSIDOR, and DRIVE data sets to evaluate network performance.

#### Internal training and validation of EyePACS data

**Fig. 5:**
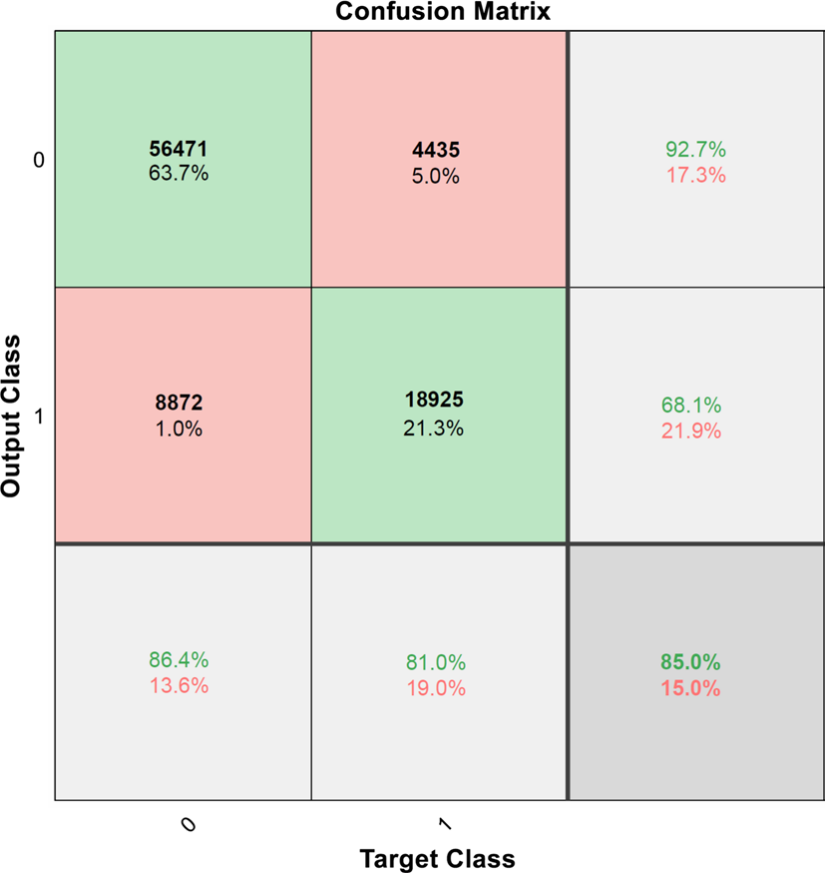
Confusion matrix for the training and validation sets for DLM1 model

*Deep learning module 1 (DLM1)*: First, we split the EyePACS dataset (88,702 images) into training arrays (70,944 images, 80%) and validation arrays (17,758 images, 20%) with a batch size of 32 images. Each epoch underwent approximately 2217 iterations (70,944/32). Training was run for 36 epochs, and a maximum of 79,812 iterations was obtained in 33,415 min of training time. The primary outcome measures of accuracy, sensitivity, specificity, and precision for the classification scores were 85.00%, 84.89%, 85.29%, 94.17%, respectively. The final trained network DLM1 achieved 85.0% accuracy for the trained and validated set and the confusion matrix in Fig. 5.

**Fig. 6:**
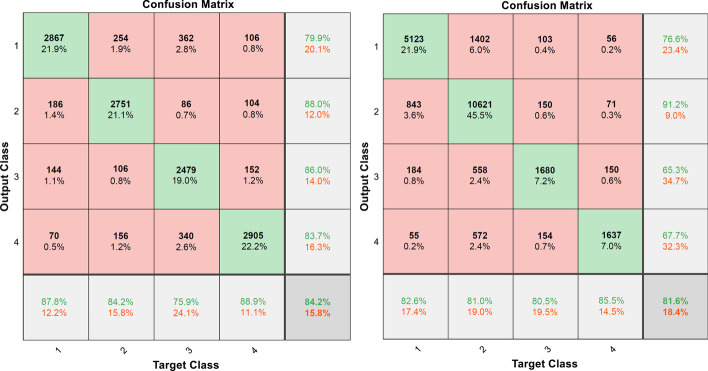
Confusion matrix for the test set (left) and EyePACs database (right) for DLM2 model

*Deep learning module 2 (DLM2)*: Then, we used SMOTE to train the model by using the EyePACS data set for four disease severity classifications. The DR level 1 to 4 (respectively 13,153, 13,153, 13,153 and 13,153) images are rotated, zoomed, and translated to increase the number of images through the SMOTE method. At the beginning of training, we divide all the data into three subsets: training set (31,568 images, 60%), validation set (10,522 images, 20%) and test set (10,522 images, 20%). The final trained network had an accuracy over test data is 84.19%. The confusion matrix in a 4-class classification for the test set and EyePACs database for DLM2 model is shown in Fig. 6. Table 1 reveals the sensitivity, specificity, accuracy, Matthews correlation coefficient and total accuracy of the DLM2 model in the DR 1–4 classification. According to Table 1, in the DR 1–4 classification, its sensitivity and specificity have good performance.

**Table 1:** Sensitivity, specificity, accuracy, Matthews correlation coefficient and total accuracy of DR level 1 to 4 for the DLM2 model

	Sensitivity (%)	Specificity (%)	Precision (%)	Matthews correlation coefficient
1: mild NPDR	82.56	90.90	76.65	0.7179
2: moderate NPDR	80.75	89.57	90.89	0.6976
3: severe NPDR	80.50	95.81	65.32	0.6953
4: proliferative DR	85.53	96.36	67.70	0.7373
Total accuracy is 81.60%				

*Deep learning module (DLM)*: In order to complete the final trained network, we combine the DLM1 and DLM2 and had an accuracy of 83.93% for DR 0–4 classification.

#### External Test of DIARETDB0, DIARETDB1, eOphtha, MESSIDOR, and DRIVE data

After internal validation, the general deep learning module for detecting DR was validated externally by using 130 images from the DIARETDB0 data set, 89 images from the DIARETDB1 data set, 463 images from the eOphtha data set, 1251 images from the MESSIDOR data set, and 40 images from the DRIVER data set that were not included in the EyePACS data set. To enable accurate validation, external validation was performed without alterations to the general deep learning model for DR detection. The results are summarized in Table 2. The outcome measures of accuracy, sensitivity, specificity, and precision for the classification of the DIARETDB0 images were 85.38%, 100%, 82.73%, and 51.28%, respectively. The same outcome measures for the classification of the DIARETDB1 images were 84.27%, 100%, 83.53%, and 22.22%, respectively. Those for the classification of the eOphtha images were 85.75%, 89.18%, 81.03%, and 86.59%, respectively. Those for the classification of the MESSIDOR images were 86.73%, 91.39%, 83.12%, and 80.74%, respectively. Finally, those for the classification the DRIVE images were 92.5%, 93.94%, 85.71%, and 96.88%, respectively.

**Table 2:** The performance of the two-stage method for no-DR and DR categories

DataBase	Accuracy (%)	Sensitivity (%)	Specificity (%)	Precision (%)
Train & validation set				
EyePACs	85.00	84.89	85.29	94.17
Test set				
DIARETDB0	85.38	100	82.73	51.28
DIARETDB1	84.27	100	83.53	22.22
e-ophtha	85.75	89.18	81.03	86.59
MESSIDOR	86.73	91.39	83.12	80.74
DRIVER	92.50	93.94	85.71	96.88

### 1-stage method (conventional study).

We used the NASNet-Large deep CNN to pretrain the model, which contained EyePACS images with no DR (65,343 sheets), mild NPDR (6205 sheets), moderate NPDR (13,153 sheets), severe NPDR (2087 sheets) and proliferative DR (1914 sheets). In order to balance the number of DR images to achieve effective feature deep learning, the DR level 1 to 4 (respectively 16,335, 16,335, 16,335 and 16,335) images are rotated, zoomed, and translated to increase the number of images through the SMOTE method. The final trained network had an accuracy of 89.79% in classifying DR into five categories of severity.

To enable accurate validation, external testing was performed without alterations to the general deep learning model for DR detection. The results are summarized in Table 3. The outcome measures of accuracy, sensitivity, specificity, and precision for the classification of the DIARETDB0 images were 61.54%, 100%, 54.55%, and 28.57%, respectively. The same outcome measures for the classification of the DIARETDB1 images were 47.19%, 75.00%, 45.88%, and 6.12%, respectively. Those for the classification of the eOphtha images were 67.82%, 54.10%, 86.67%, and 84.80%, respectively. Those for the classification of the MESSIDOR images were 59.39%, 99.81%, 28.09%, and 51.81%, respectively. Finally, those for the classification the DRIVE images were 87.50%, 100%, 28.57%, and 86.84%, respectively.

**Table 3:** The performance of the one-stage method for no-DR and DR categories

DataBase	Accuracy (%)	Sensitivity (%)	Specificity (%)	Precision (%)
Train & validation set				
EyePACs	89.79	91.20	85.87	94.75
Test set				
DIARETDB0	61.54	100	54.55	28.57
DIARETDB1	47.19	75.00	45.88	06.12
e-ophtha	67.82	54.10	86.67	84.80
MESSIDOR	59.39	99.81	28.09	51.81
DRIVER	87.50	100	28.57	86.84

## Summary

We adopted a 2-stage training method to classify the data into normal and abnormal in deep learning module 1, and then used the SMOTE in deep learning module 2 to solve the problem of imbalance training data. This study used a 2-stage training method initially to reduce oversampling, early stopping and data dividing methods to avoid overfitting. In a conventional study using the 1-stage training method, the SMOTE will generate 238,013 synthetic samples under an unbalanced data set. This research proposes a 2-stage training method to generate 29,253 images to meet the requirements of a balanced data set. Comparing the number of the above two synthetic samples, the method proposed reduces the number of images by nearly 87.7% and reduces the possibility of overfitting.

According to our experiments, 2-stages training method is better than the 1-stage training method and the diagnostic model’s prediction accuracy for unseen images (test set) was approximately 85.38%–86.73% similar to the 2015 the team (Min-Pooling) that won the championship in the Kaggle Diabetic Retinopathy Detection competition in 2015. Therefore, this diagnostic model can be used for DR detection with images provided by different hospitals or research institutions. It can be used as a general deep learning model for detecting DR.

## Discussion

EyePACS provided DR images for this experiment. Images in the dataset were obtained using different camera makes and models, which may affect the visual appearance of the left and right lenses. Furthermore, image and label noise, such as artifacts, blurred focus, underexposure, or overexposure may be present. Therefore, image processing techniques must be used to extract useful features from these images for further analysis.

**Fig. 7:**
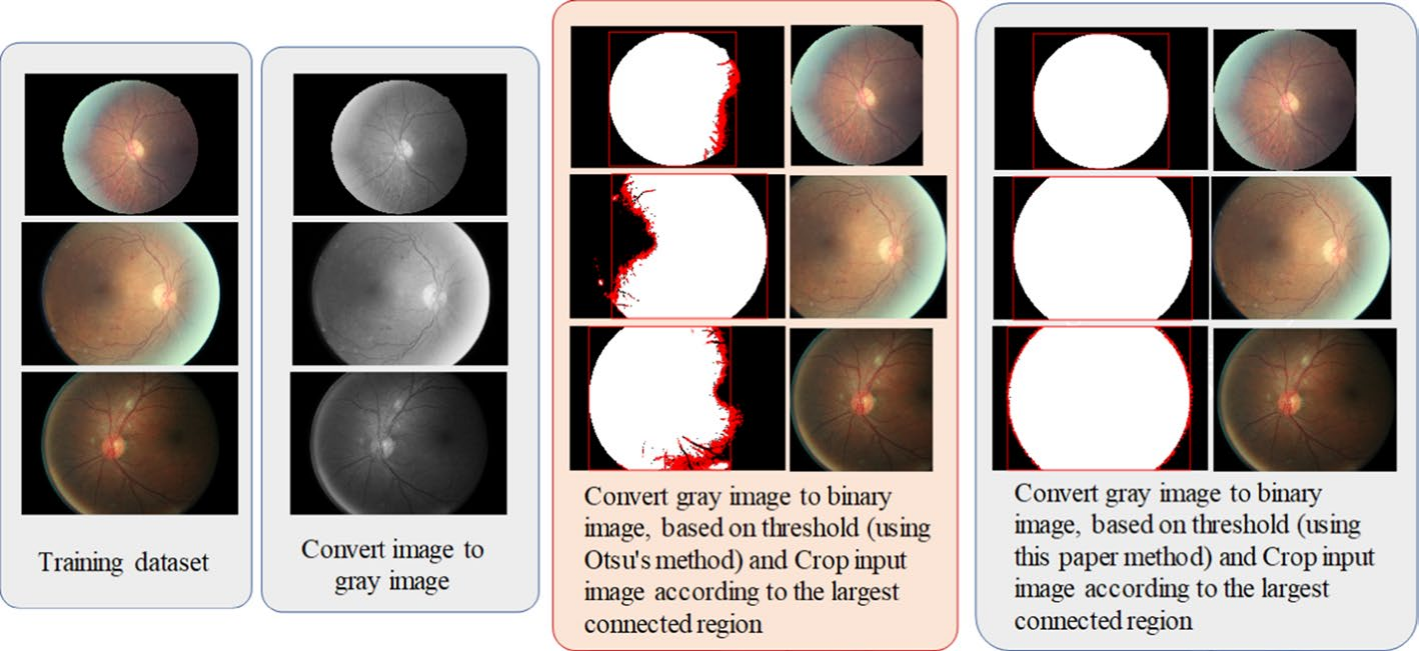
Examples of image preprocessing

The experiment revealed that the failure rate of image cropping at the edge of the eyeball is high. After several tests, a GRAYTHRESH function [33] with a gray value of + 200 was used, and the obtained binarized images could be used to perfectly detect the edge of the eyeball. This step made the available recognition rate even more crucial. In the NASNet model, weights were pretrained using ImageNet. The default input size for the NASNet-Large model was 331 × 331 pixels. However, according to the NASNet-Large model, training larger images (e.g., 3504 × 2336 pixels) that are adjusted to 331 × 331 pixels would cause image distortion; therefore, we used the fixed radius method for image cropping during image preprocessing to ensure that the image outline was not deformed. Figure 7 presents examples of preprocessing.

Increased convergence can be achieved with a large minibatch size. In addition to the effect on computational throughput, minibatch size affects training accuracy for resource use. To investigate this, we trained the NASNet-Large CNN with the EyePACS data set for 54 epochs, and three NVIDIA TESLA P100 16-GB GPUs were used during deep learning to explore the effect of different minibatch sizes on the accuracy rate. Table 4 presents the verification accuracies of these runs. The minibatch accuracy of 32 is significantly better than the minibatch accuracy of 16. In addition, 88,702 data points in the EyePACS training set, 88,949,818 NASNet-Large parameters, and 1244 layers of depth were used. The experiment confirmed that three TESLA P100 16-GB GPUs should use minibatches of less than 32. Minibatches of more than 32 cause data loss from GPU memory.

**Table 4:** Performance of the NASNet-Large deep CNN with different minibatch configurations in the first iteration

GPU	Mini-batch					
	8	12	16	24	32	36
NVIDIA GTX-1080Ti-11 GB GPU × 2	70.5%	71.5%	–	–	–	–
NVIDIA Tesla-P100-16 GB GPU × 3	70.5%	71.5%	72.5%	73.5%	75.4%	–

In this article, for the multi-category diagnosis of diabetic retinopathy, we use a two-stage training model architecture. In the first stage, we do not use artificial methods to increase the number of data sets, and integrate the original images of 2, 3, and 4 categories into 1 category to obtain a larger number of 1 category. The no-DR and DR diagnostic models can avoid over-fitting problems caused by over-sampling. Finally, the sensitivity and specificity of the tested model rarely ignore true positives, and rarely identify other things that are not the target of the test as positive. This approach of this two-stage training model significantly alleviates the problem of category imbalance and improves the generalization performance of the deep learning module.

## Conclusions

In this experiment, a general deep learning model for detecting DR was developed for application in all DR databases. Our research provides a straightforward method of addressing imbalance in DR databases that can be applied to other medical images in the future.

## Data Availability

The datasets generated and analyzed during the current study are available in the Kaggle repository, https://www.kaggle.com/c/diabetic-retinopathy-detection.

## Abbreviations

DR: Diabetic retinopathy
SMOTE: Synthetic minority oversampling technique
CNN: Convolutional neural network
GPU: Graphics processing unit
CUDA: Compute unified device architecture
SDGM: Skip deep generative model
ADAM: Adaptive data momentum
DLM: Deep learning module
DLM1: Deep learning module 1
DLM2: Deep learning module 2

## References

[1] Abràmoff MD, Lavin PT, Birch M, Shah N, Folk JC (2018). Pivotal trial of an autonomous AI-based diagnostic system for detection of diabetic retinopathy in primary care offices. NPJ Digital Med.

[2] Ting DSW, Peng L, Varadarajan AV (2019). Deep learning in ophthalmology: the technical and clinical considerations. Prog Retin Eye Res.

[3] Bellemo V, Lim G, Rim TH (2019). Artificial intelligence screening for diabetic retinopathy: the real-world emerging application. Curr Diab Rep.

[4] Du XL, Li WB, Hu BJ (2018). Application of artificial intelligence in ophthalmology. Int J Ophthalmol.

[5] Grewal PS, Oloumi F, Rubin U, Tennant MTS (2018). Deep learning in ophthalmology: a review. Can J Ophthalmol.

[6] Lam C, Yu C, Huang L, Rubin D (2018). Retinal lesion detection with deep learning using image patches. Invest Ophthalmol Vis Sci.

[7] Tsao HY, Chan PY, Su ECY (2018). Predicting diabetic retinopathy and identifying interpretable biomedical features using machine learning algorithms. BMC Bioinform.

[8] Kaggle, Inc. Diabetic Retinopathy Detection. https://www.kaggle.com/c/diabetic-retinopathy-detection . Accessed 20 April 2020.

[9] Mansour RF (2018). Deep-learning-based automatic computer-aided diagnosis system for diabetic retinopathy. Biomed Eng Lett.

[10] Fernandez A, Garca S, Herrera F, Chawla NV (2018). SMOTE for learning from imbalanced data: progress and challenges, Marking the 15-year Anniversary. J Artif Intell Res.

[11] Chawla NV, Bowyer KW, Hall LO, Kegelmeyer WP (2002). SMOTE: synthetic minority over-sampling technique. J Artif Intell Res.

[12] White C, Ismail HD, Saigo H, KC DB (2017). CNN-BLPred: a Convolutional neural network based predictor for β-Lactamases (BL) and their classes. BMC Bioinform.

[13] Wu Z, Guo Y, Lin W, Yu S, Ji Y (2018). A weighted deep representation learning model for imbalanced fault diagnosis in cyber-physical systems. Sensors (Basel).

[14] Lin GM, Chen MJ, Yeh CH, Lin YY, Kuo HY, Lin MH, Chen MC, Lin SD, Gao Y, Ran A, Cheung CY. Transforming retinal photographs to entropy images in deep learning to improve automated detection for diabetic retinopathy. J Ophthalmol. 2018;2018:2159702. 10.1155/2018/2159702 PMC6151683 30275989

[15] Moss HB, Leslie DS, Rayson P. Using J-K fold cross validation to reduce variance when tuning NLP models. In: Proceedings of the 27th international conference on computational linguistics. 2018;1–12.

[16] Jiang J, Liu X, Zhang K, Long E, Wang L, Li W, Liu L, Wang S, Zhu M, Cui J, Liu Z, Lin Z, Li X, Chen J, Cao Q, Li J, Wu X, Wang D, Wang J, Lin H (2017). Automatic diagnosis of imbalanced ophthalmic images using a cost-sensitive deep convolutional neural network. Biomed Eng Online.

[17] Mitra A, Banerjee PS, Roy S, Roy S, Setua SK (2018). The region of interest localization for glaucoma analysis from retinal fundus image using deep learning. Comput Methods Programs Biomed.

[18] Zago GT, Andreão RV, Dorizzi B, Salles EOT (2018). Retinal image quality assessment using deep learning. Comput Biol Med.

[19] Khojasteh P, Júnior LAP, Carvalho T, Rezende E, Aliahmad B, Papa JP, Kumar DK (2019). Exudate detection in fundus images using deeply-learnable features. Comput Biol Med.

[20] Islam SS, Dey EK, Tawhid MNA, Hossain BM (2017). A CNN based approach for garments texture design classification. Adv Technol Innov.

[21] Quellec G, Charrière K, Boudi Y, Cochener B, Lamard M (2017). Deep image mining for diabetic retinopathy screening. Med Image Anal.

[22] Abràmoff MD, Lou Y, Clarida W, Amelon R, Folk JC, Niemeijer M (2016). Improved automated detection of diabetic retinopathy on a publicly available dataset through integration of deep learning. Invest Ophthalmol Vis Sci.

[23] Cao Y, Montgomery S, Ottosson J, Naslund E, Stenberg E (2020). Deep learning neural networks to predict serious complications after bariatric surgery: analysis of scandinavian obesity surgery registry data. JMIR Med Inform.

[24] Wang S, Li Z, Chao W, Cao Q. Applying adaptive over-sampling technique based on data density and cost-sensitive SVM to imbalanced learning. In: The 2012 international joint conference on neural networks (IJCNN), Brisbane, Australia, June 10–15 (2012).

[25] Cuadros J, Bresnick G (2009). EyePACs: an adaptable telemedicine system for diabetic retinopathy screening. J Diabetes Sci Technol.

[26] Zoph B, Vasudevan V, Shlens J, Le QV. Learning transferable architectures for scalable image recognition. In: IEEE conference on computer vision and pattern recognition. 2018;8697–8710.

[27] Kauppi T, Kalesnykiene V, Kamarainen JK, Lensu L, Sorri I, Uusitalo H, Kälviäinen H, Pietilä J. DIARETDB0: evaluation database and methodology for diabetic retinopathy algorithms; machine vision and pattern recognition research group. Lappeenranta University of Technology, Lappeenranta, 2006;73.

[28] Kauppi T, Kalesnykiene V, Kamarainen JK, Lensu L, Sorri I, Raninen A, Voutilainen R, Uusitalo H, Kalviainen H, Pietila J. The diaretdb1 diabetic retinopathy database and evaluation protocol. In: Proceedings of the British machine vision conference. BMVA Press. 2007;15.1–15.10.

[29] Decencire E, Cazuguel G, Zhang X, Thibault G, Klein JC, Meyer F, Marcotegui B, Quellec G, Lamard M, Danno R, Elie D, Massin P, Viktor Z, Erginay A, La B, Chabouis A (2013). Teleophta: machine learning and image processing methods for teleophthalmology. IRBM.

[30] MESSIDOR: MESSIDOR stands for methods to evaluate segmentation and indexing techniques in the field of retinal ophthalmology. http://www.adcis.net/en/third-party/messidor/. Accessed 20 April 2020.

[31] Staal JJ, Abramoff MD, Niemeijer M, Viergever MA, Ginneken BV (2004). Ridge based vessel segmentation in color images of the retina. IEEE Trans Med Imaging.

[32] Pretrained Deep Neural Networks. https://www.mathworks.com/help/deeplearning/ug/pretrained-convolutional-neural-networks.html. Accessed 20 April 2020.

[33] Otsu N (1979). A threshold selection method from gray-level histograms. IEEE Trans Syst Man Cybernet.

